# Supplementary material for: Wetting Characteristics of Nanosilica-Poly (acrylic acid) Transparent Anti-Fog Coatings
Source: Polymers (Basel). 2022 Nov 1;14(21):4663. doi: 10.3390/polym14214663 (PMC9655888; doi:10.3390/polym14214663)
Supplement: Supplementary file 1 [file polymers-14-04663-s001.zip › polymers-1954550-supplementary.pdf]

Supporting Information for

Wetting Characteristics of Nanosilica-Poly (acrylic acid) Trans-parent Anti-fog Coatings

Sevil Turkoglu[1], Jinde Zhang[1], Hanna Dodiuk[2], Samuel Kenig[2], Jo Ann Ratto[3], Joey Mead[1*]

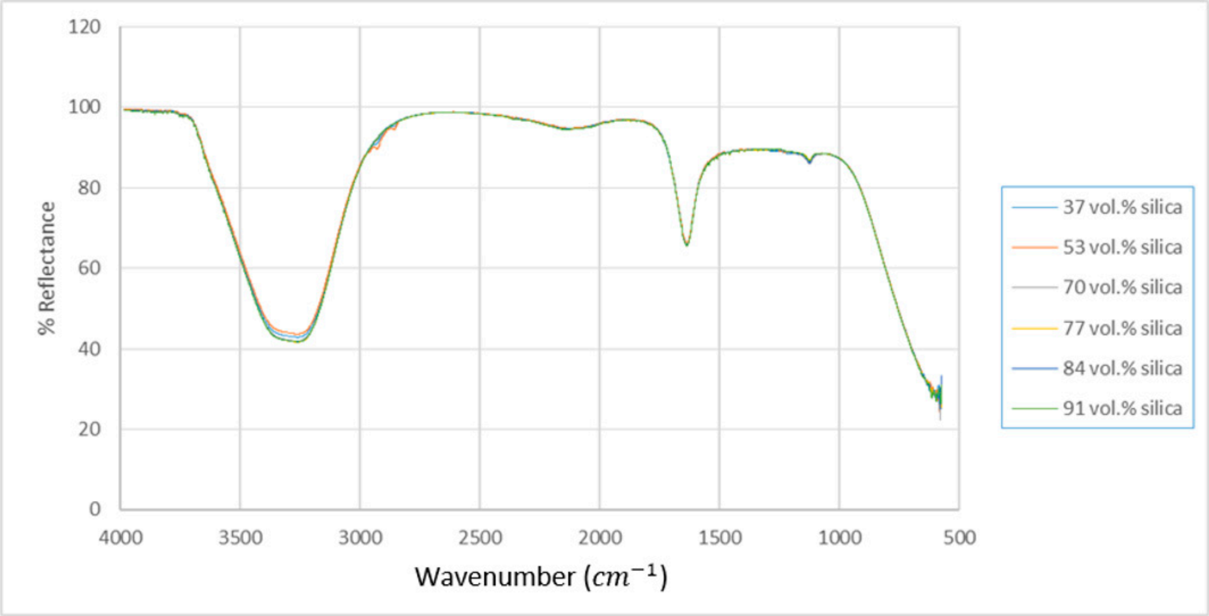

Figure S1. FTIR spectrum of several samples

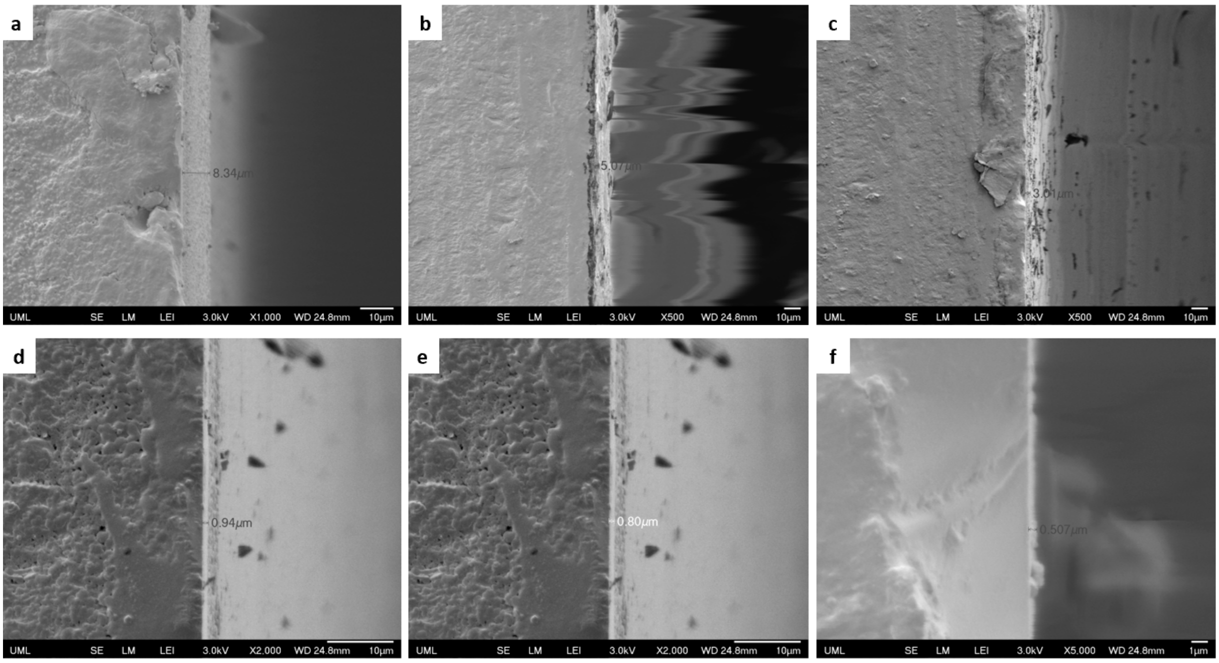

Figure S2. SEM cross section images of coatings with particle loading (vol. %) of a) 37, b) 53, c) 70, d) 77, e) 84, f) 91

Table S1. Coating thickness values

Particle loading (% vol.)	37	53	70	77	84	91
Coating Thickness (µm)	8.34±0.7	5.07±0.9	3.01±0.6	0.94±0.3	0.80±0.4	0.50±0.3

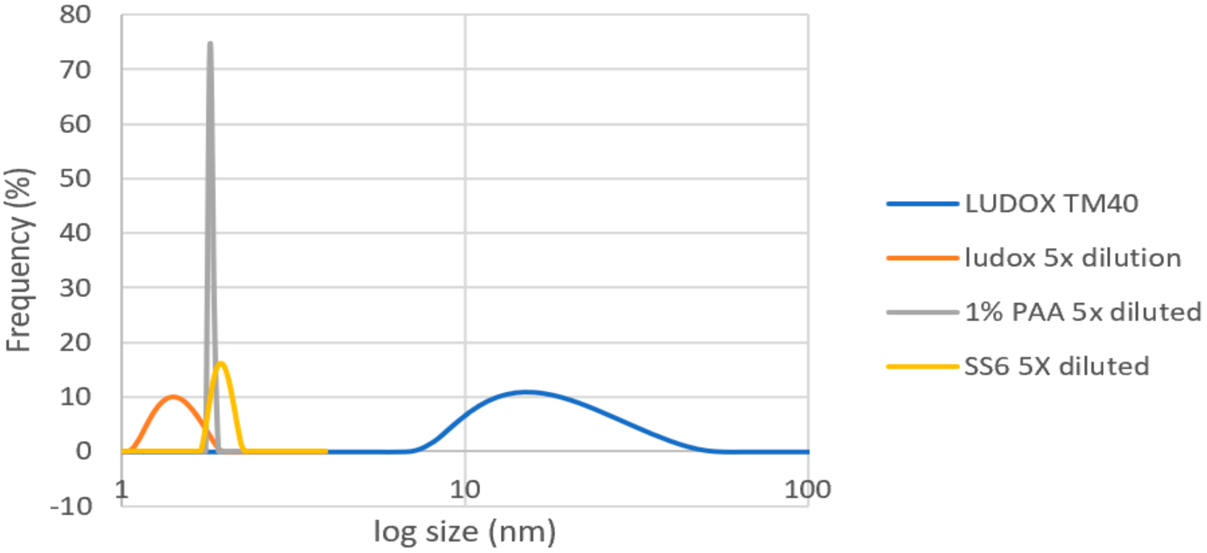

Figure S3. Size distribution curves

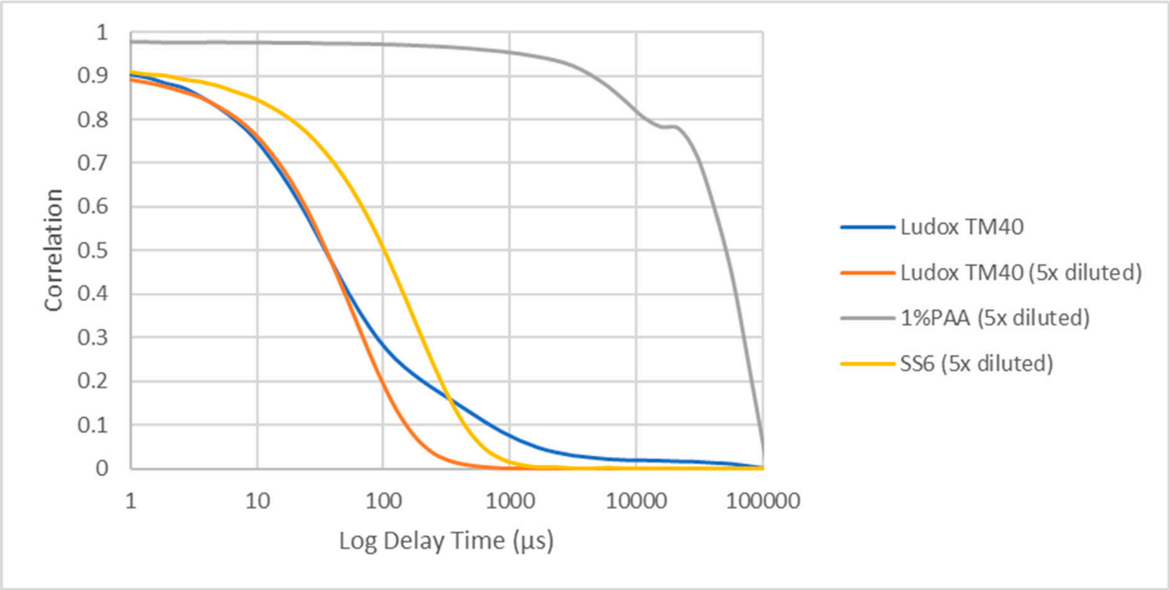

Figure S4. Correlation function vs delay time

Table S2. FTIR peak assignments

**Wavenumber (cm^{-1})**	**Tentative assignment of functional group**
3255	OH stretching and hydrogen bonding
1074	Si–O–Si stretching
767	Si–O bending
1635	C=O stretching
1122	C-O stretching vibrations
2135	CO_2
1555	C=C stretching
900	C=C bending
821	C-H bending

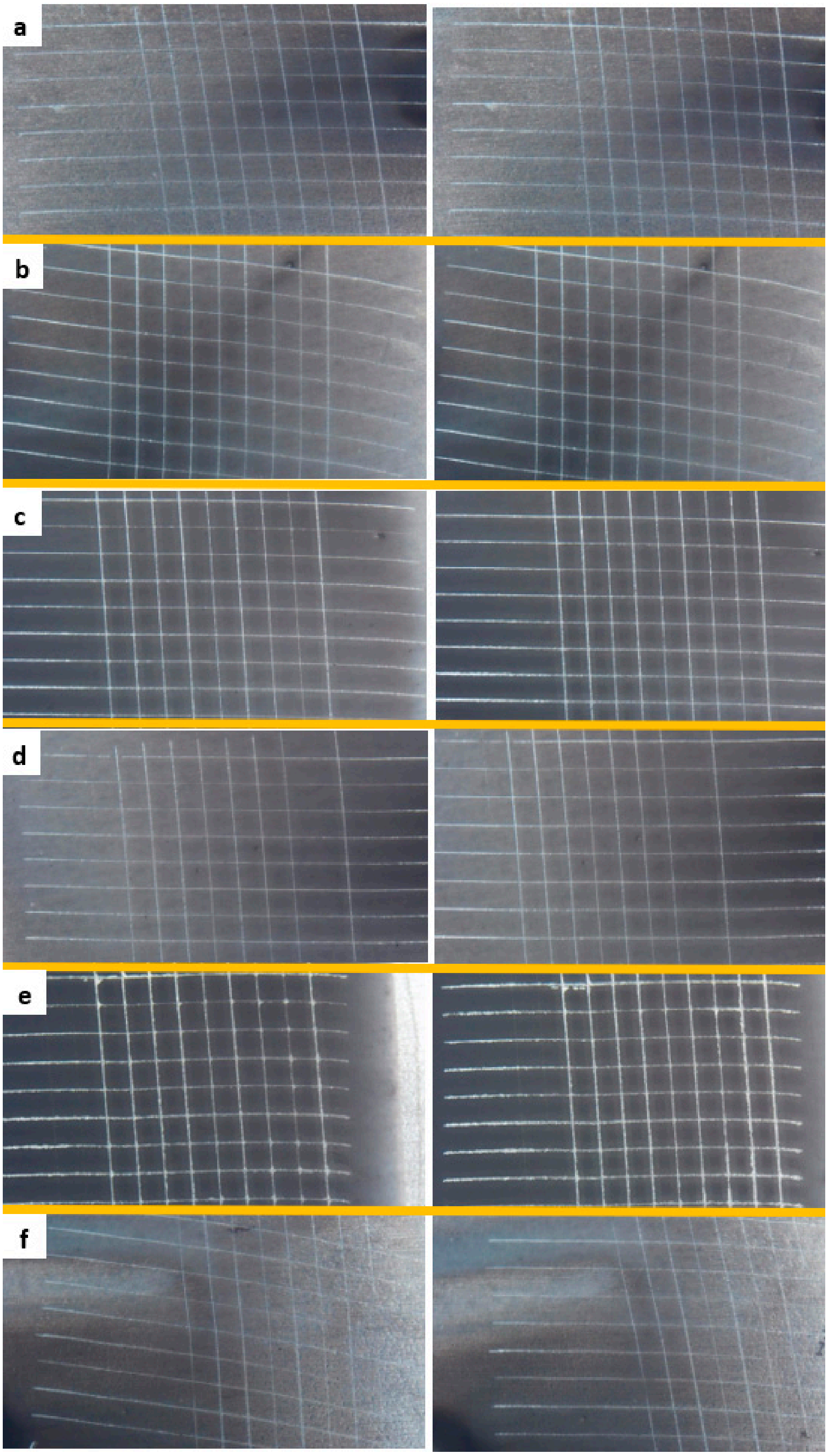

Figure S5. Optical microscopy images of coatings with particle loading (vol. %) of a) 37, b) 53, c) 70, d) 77, e) 84, f) 91 before (left) and after (right) adhesion test
